# Prenatal metal exposure, cord blood DNA methylation and persistence in childhood: an epigenome-wide association study of 12 metals

**DOI:** 10.1186/s13148-021-01198-z

**Published:** 2021-11-19

**Authors:** Anne K. Bozack, Sheryl L. Rifas-Shiman, Brent A. Coull, Andrea A. Baccarelli, Robert O. Wright, Chitra Amarasiriwardena, Diane R. Gold, Emily Oken, Marie-France Hivert, Andres Cardenas

**Affiliations:** 1 Division of Environmental Health Sciences, School of Public Health, University of California Berkeley, 2121 Berkeley Way, Room 5302, Berkeley, CA 94720 USA; 2 Division of Chronic Disease Research Across the Lifecourse, Department of Population Medicine, Harvard Medical School and Harvard Pilgrim Health Care Institute, Boston, MA USA; 3 Department of Biostatistics, Harvard T.H. Chan School of Public Health, Harvard University, Boston, MA USA; 4 Department of Environmental Health Sciences, Mailman School of Public Health, Columbia University, New York City, NY USA; 5 Department of Environmental Medicine and Public Health and Institute for Exposomic Research, Icahn School of Medicine at Mount Sinai, NY New York City, USA; 6 Channing Division of Network Medicine, Department of Medicine, Brigham and Women’s Hospital, Boston, MA USA; 7 Department of Environmental Health, Harvard T.H. Chan School of Public Health, Harvard University, Boston, MA USA; 8 Harvard Medical School, Boston, MA USA; 9 Diabetes Unit, Massachusetts General Hospital, Boston, MA USA; 10 Center for Computational Biology, University of California, Berkeley, CA USA

**Keywords:** DNA methylation, EWAS, Manganese, Metals, Prenatal exposure

## Abstract

**Background:**

Prenatal exposure to essential and non-essential metals impacts birth and child health, including fetal growth and neurodevelopment. DNA methylation (DNAm) may be involved in pathways linking prenatal metal exposure and health. In the Project Viva cohort, we analyzed the extent to which metals (As, Ba, Cd, Cr, Cs, Cu, Hg, Mg, Mn, Pb, Se, and Zn) measured in maternal erythrocytes were associated with differentially methylated positions (DMPs) and regions (DMRs) in cord blood and tested if associations persisted in blood collected in mid-childhood. We measured metal concentrations in first-trimester maternal erythrocytes, and DNAm in cord blood (*N* = 361) and mid-childhood blood (*N* = 333, 6–10 years) with the Illumina HumanMethylation450 BeadChip. For each metal individually, we tested for DMPs using linear models (considered significant at FDR < 0.05), and for DMRs using *comb-p* (Sidak *p* < 0.05). Covariates included biologically relevant variables and estimated cell-type composition. We also performed sex-stratified analyses.

**Results:**

Pb was associated with decreased methylation of cg20608990 (*CASP8*) (FDR = 0.04), and Mn was associated with increased methylation of cg02042823 (*A2BP1*) in cord blood (FDR = 9.73 × 10^–6^). Both associations remained significant but attenuated in blood DNAm collected at mid-childhood (*p* < 0.01). Two and nine Mn-associated DMPs were identified in male and female infants, respectively (FDR < 0.05), with two and six persisting in mid-childhood (*p* < 0.05). All metals except Ba and Pb were associated with ≥ 1 DMR among all infants (*Sidak p* < 0.05). Overlapping DMRs annotated to genes in the human leukocyte antigen (HLA) region were identified for Cr, Cs, Cu, Hg, Mg, and Mn.

**Conclusions:**

Prenatal metal exposure is associated with DNAm, including DMRs annotated to genes involved in neurodevelopment. Future research is needed to determine if DNAm partially explains the relationship between prenatal metal exposures and health outcomes.

**Supplementary Information:**

The online version contains supplementary material available at 10.1186/s13148-021-01198-z.

## Background

Essential trace metals are critical for fetal development [1]. Copper (Cu) [2], magnesium (Mg) [3], manganese (Mn) [4], selenium (Se) [5], and zinc (Zn) [6] are involved in many biological processes and serve as cofactors or allosteric components to enzymes. Low maternal status of essential metals has been associated with adverse birth outcomes including preterm birth [7], decreased birth weight, and growth restriction [4], as well as infant and child health outcomes including neurodevelopment, cognitive function [8–10], and increased risk for infections [10].

Environmental exposure to harmful heavy metals and metalloids, collectively referred to here as “metals,” from natural and anthropogenic sources is also a public health concern. The toxicity of some heavy metals can lead to adverse health outcomes through various biological mechanisms including oxidative stress, disruption of cell membrane potential and homeostasis, and competitive binding to proteins [11, 12]. Higher levels of non-essential metals including arsenic (As), cadmium (Cd), and lead (Pb) have been associated with risk of low birth weight [13–15]. In addition, As, Cd, mercury (Hg), and Pb are potent neurotoxicants, and prenatal exposure to these metals has been associated with decreased measures of neurodevelopment and cognitive function in children [16–19]. Essential metals may also have nonlinear associations with child health. For example, maternal Mn has been shown to have an inverse U-shaped relationship with neurodevelopment in infants, with low and high levels associated with poor mental and psychomotor development [8]. Additionally, sex-specific associations for metals and health, including metal uptake and retention, have been reported, highlighting the need to study sex-specific associations [20, 21]. Specifically, sex-specific associations have been reported with early-pregnancy maternal metal concentrations and birth outcomes in the Project Viva cohort [22].

Changes in epigenetic markers may be involved in the biological pathways, or serve as biomarkers, relating metal exposure to infant and child health. Embryonic development is a time of rapid epigenetic reprogramming, which may impact health outcomes later in life. As proposed by the developmental origins of health and disease (DOHaD) hypothesis [23], pre- and postnatal exposures that occur during critical developmental periods may affect disease phenotypes through epigenetic mechanisms including DNA methylation (DNAm) reprogramming [24]. Prenatal maternal exposure to a range of environmental contaminants including some metals has been associated with alterations in global and locus-specific DNAm [25].

For selected metals, there is ample evidence of epigenetic dysregulation. For example, multiple epigenome-wide association studies (EWAS) of prenatal As exposure have been conducted, including populations with both low [26, 27] and high exposure levels [28–30]; however, a common epigenetic signature of prenatal As exposure has not been identified, but rather multiple cohort-specific signals. Fewer EWAS of other prenatal metal exposures have been conducted. For Pb, two EWAS have identified varying signatures across populations. An EWAS prenatal Pb exposure and cord blood DNAm conducted in a cohort in Mexico did not identify significant associations between Pb and locus-specific DNAm [31]. However, an EWAS conducted among mother–infant pairs in the United States (US)-based Project Viva cohort (*N* = 268) identified four CpGs associated with second-trimester maternal red blood cell (RBC) Pb concentrations among infants overall, with 38 CpGs among female and two among male infants in sex-stratified analyses (FDR < 0.05) [32]. Also, within Project Viva, second-trimester maternal RBC Hg concentrations were associated with methylation of one CpG among infants overall and of one CpG among males (*N* = 321; *p*_Bonferroni_ < 0.05) [33].

Considering the limited epidemiological research on associations between prenatal metal exposures and epigenetic dysregulation, in this study we investigate the extent to which prenatal metals measured in first-trimester maternal RBCs are associated with DNAm profiles in cord blood and compare associations across metals. Leveraging data and samples from Project Viva, a pre-birth cohort with relatively low levels of metal exposures, we analyzed associations between prenatal exposure to 12 metals [As, barium (Ba), Cd, chromium (Cr), cesium (Cs), Cu, Hg, Mg, Mn, Pb, Se, Zn] and differentially methylated positions (DMPs) and regions (DMRs) in cord blood. The current research expands upon previous analyses conducted in Project Viva by investigating multiple metals measured during the first trimester, reflecting exposure during embryonic, rather than fetal, development. We also investigated sex-specific associations and persistence of observed associations in mid-childhood.

## Results

### Participant characteristics

DNAm data were available for 361 (Additional file 1: Figure S1) and 333 mother–infant pairs at birth and in mid-childhood, respectively, and a total of 189 mother–infant pairs had both cord blood and mid-childhood DNAm data. Characteristics of mother–infant pairs and maternal blood metal concentrations are provided in Table 1. Among participants with cord blood DNAm data, the median (IQR) maternal age at enrollment was 32.4 (29.7, 35.9) years. The majority of mothers were college graduates and had household incomes > $70,000 per year (70.6% and 60.4%, respectively). Approximately half of infants were female (46.8%), 70.4% were White, and 11.4% were Black. Spearman correlations between metals are presented in Additional file 1: Figure S2. The greatest correlations were observed between Cu and Zn (*r* = 0.56), As and Hg (*r* = 0.50), Cu and Mg (*r* = 0.45), and Mg and Se (*r* = 0.44) (*p* < 0.05). There were no significant differences in baseline characteristics or prenatal maternal blood metal concentrations between mother–infant pairs with cord blood or mid-childhood DNAm data (*p* > 0.10).

**Table 1:** Characteristics of Project Viva mother–infant pairs with cord blood and mid-childhood samples

	Cord blood (*N* = 361)		Mid-childhood (*N* = 333)^a^	
	*N* or median	% or IQR	*N* or median	% or IQR
*Maternal characteristics*				
Age at enrollment (years), median (IQR)	32.4	(29.7, 35.9)	32.9	(29.7, 36.2)
College graduate	255	70.6%	234	70.3%
Smoking during pregnancy	41	11.4%	38	11.4%
Annual household income > $70,000	218	60.4%	206	61.9%
Nulliparous	177	49.0%	150	45.0%
Prenatal BMI, median (IQR)	23.5	(21.3, 27.2)	23.5	(21.5, 26.6)
Red blood cell concentration (ng/g), median (IQR)				
As	0.86	(0.46, 1.5)	0.81	(0.44, 1.64)
Ba	3.15	(2.06, 5.95)	3.10	(1.96, 5.57)
Cd	0.39	(0.27, 0.56)	0.40	(0.27, 0.55)
Cr	1.31	(0.86, 1.91)	1.37	(0.87, 2.16)
Cs	2.53	(2.02, 3.14)	2.48	(1.94, 3.14)
Cu	562	(517, 620)	560	(513.0, 619.0)
Hg^b^	3.19	(1.69, 6.68)	3.26	(1.85. 5.89)
Mg	41,500	(37,100, 46,400)	41,000	(36,600, 46,000)
Mn	15.80	(13.10, 19.70)	15.70	(12.80, 19.80)
Pb	18.10	(13.90, 23.80)	18.00	(14.00, 19.00)
Se	249	(222, 279)	247	(222, 274)
Zn	10,300	(9,380, 11,600)	10,300	(9,280, 11,500)
*Infant characteristics*				
Female	169	46.8%	158	47.4%
Gestational age (weeks), median (IQR)	40	(39.0, 40.9)	39.9	(38.9, 40.6)
Birth weight z-score	0.19	(− 0.35, 0.90)	0.24	(− 0.35, 0.97)
Race/ethnicity				
White	254	70.4%	221	66.4%
Black	41	11.4%	52	15.6%
Hispanic	20	5.5%	17	5.1%
Asian	8	2.2%	9	2.7%
More than one race or other	38	10.5%	34	10.2%
Age at mid-childhood blood draw (years), median (IQR)	–	–	7.66	(7.38, 8.25)

^a^A total of 189 mother–infant pairs had both cord blood and mid-childhood DNAm data

^b^Cord blood: *N* = 358; mid-childhood: *N* = 326; data are complete for all other variables

In linear models adjusted for infant sex, race, gestational age, maternal age, pre-pregnancy BMI, education, household income, and smoking, prenatal Cu exposure was associated with CD4 + T cell proportions estimated using the Houseman regression calibration method. For every doubling of Cu concentration, there was an increase of 3.25% in the proportion of estimated CD4 + T cells (*p* = 0.037) (Additional file 1: Table S1). There was a trend toward positive associations between Cd and CD4 + T cells and between Hg and monocytes, although these associations did not achieve statistical significance (*B* = 0.63, *p* = 0.05 and *B* = 0.23, *p* = 0.05, respectively).

### Differentially methylated positions

**Fig. 1:**
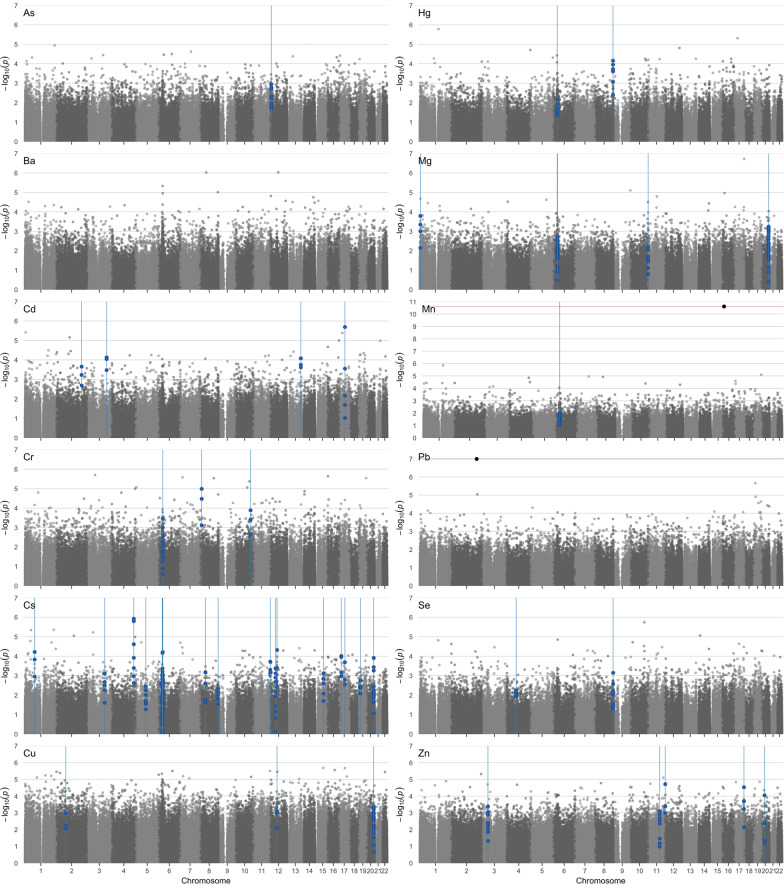
Manhattan plots for epigenome-wide associations between prenatal metal exposure and cord blood DNA methylation. Models of log_2_-transformed prenatal metal concentrations adjusted for infant sex, race/ethnicity, gestational age, nulliparous, maternal age at enrollment, pre-pregnancy BMI, education, smoking, household income, and estimated cord blood cell-type proportions. DMRs are indicated by blue lines; the FDR cutoffs for Mn and Pb are indicated by red lines

Manhattan and Q-Q plots for each metal in fully adjusted models for cord blood DNAm are shown in Fig. 1 and Additional file 1: Figure S3, respectively; λ values are presented in Additional file 1: Table S2. Full results of EWAS for each metal are available on the study’s GitHub repository (https://github.com/annebozack/viva_DNAm_metals) and Open Science Framework site (https://osf.io/jf5yt/). Among infants overall, prenatal exposure to Mn was associated with greater methylation of cg02042823, annotated to *A2BP1* (Table 2). Each doubling of prenatal Mn concentrations was associated with 2.65% greater methylation (95% CI: 2.07, 3.23; *p* = 2.47 × 10^–11^; FDR = 9.7327 × 10^–6^) at this CpG. Pb exposure was associated with 3.10% lower DNAm at cg20608990, annotated to *CASP8*, with each doubling of Pb concentrations (95% CI: − 4.22, − 1.97, *p* = 1.02 × 10^–7^, FDR = 0.04) (Table 2). No DMPs achieved statistical significance in analyses of other metal exposures among infants overall (FDR > 0.05), or in sensitivity analyses for As and Hg adjusting for maternal fish intake.

**Table 2:** Cord blood differentially methylated positions associated with Mn and Pb (FDR < 0.05)

CpG	Chr	Position^a^	Gene	Feature category	Cord blood				Mid-childhood		
					*p*	FDR	Median % methylation	Mean difference in % methylation (95% CI)	*p*	Median % methylation	Mean difference in % methylation (95% CI)
Mn											
All infants^b^											
cg02042823	16	6,714,429	*A2BP1*	5'UTR	2.47 × 10^–11^	9.73 × 10^–6^	98.29	2.65 (2.07, 3.23)	0.010	98.65	0.75 (0.14, 1.36)
Female^c^											
cg00954161	1	3,696,925	*LRRC47*	3'UTR	2.23 × 10^–4^	0.037	98.5	1.54 (1.08, 2.01)	0.001	98.41	1.33 (0.69, 1.98)
cg11161853	3	67,705,044	*SUCLG2*	TSS200	1.03 × 10^–6^	0.048	2.43	− 0.32 (− 0.43, − 0.22)	0.73	2.37	0.01 (− 0.06, 0.07)
cg23903787	4	3,527,371	*LRPAP1*	Body	3.13 × 10^–7^	0.038	89.44	4.60 (3.32, 5.88)	0.011	89.48	2.04 (0.79, 3.29)
cg19908812	4	164,253,006	*NPY1R*	5'UTR	9.36 × 10^–7^	0.048	1.47	− 0.30 (− 0.38, − 0.21)	0.55	2.90	− 0.03 (− 0.34, 0.27)
cg26462130	7	2,052,961	*MAD1L1*	Body	1.10 × 10^–6^	0.048	98.63	2.04 (1.67, 2.41)	0.005	98.71	0.98 (0.68, 1.28)
cg08904630	10	71,490,427	–		9.42 × 10^–7^	0.048	97.74	0.84 (0.64, 1.04)	1.27 × 10^–5^	97.93	0.48 (0.34, 0.62)
cg22799518	12	56,988,862	*RBMS2*	3'UTR	3.80 × 10^–7^	0.037	98.80	2.03 (1.62, 2.44)	0.004	98.74	− 0.20 (− 0.43, 0.03)
cg01744822	16	73,100,510	–		9.19 × 10^–7^	0.048	4.59	− 1.16 (− 1.45, − 0.86)	9.44 × 10^–5^	8.89	− 1.66 (− 2.29, − 1.03)
cg15712310	16	73,100,790	–		1.93 × 10^–7^	0.037	24.87	− 2.69 (− 3.56, − 1.81)	0.039	36.01	− 0.97 (− 1.86, − 0.07)
Male^**d**^											
cg03763518	1	150,245,044	*C1orf54*	TSS200	4.75 × 10^–8^	0.009	7.62	− 3.01 (− 3.72, − 2.30)	1.74 × 10^–4^	32.41	− 3.03 (− 4.56, − 1.49)
cg02042823	16	6,714,429	*A2BP1*	5'UTR	2.79 × 10^–10^	1.10 × 10^–4^	98.22	3.40 (2.73, 4.07)	2.34 × 10^–4^	98.65	1.82 (0.51, 3.13)
Pb											
All infants ^b^											
cg20608990	2	202,097,607	*CASP8*	TSS1500	1.02 × 10^–7^	0.040	64.54	− 3.10 (− 4.22, − 1.97)	0.008	48.64%	− 1.56 (− 2.73, − 0.40)

Differentially methylated positions (DMPs) identified using models of individual log_2_-transformed prenatal metal concentration adjusted for infant sex, race/ethnicity, gestational age (age at blood collection for mid-childhood models), nulliparous, maternal age at enrollment, pre-pregnancy BMI, education, smoking, household income, and estimated cord blood cell-type proportions (estimated in cord blood or from leukocytes in mid-childhood). Mean difference in % methylation determined using the same adjusted models for Beta-values for interpretation of results

^a^hg 19 assembly

^b^Cord blood: *N* = 361; mid-childhood: *N* = 333

^c^Female (Cord blood: N = 169; mid-childhood: *N* = 158)

^d^Male (Cord blood: *N* = 192; mid-childhood: *N* = 175)

In stratified analyses, Mn exposure was associated with nine DMPs among females [cg00954161 (*LRRC47*), cg11161853 (*SUCLG2*), cg23903787 (*LRPAP1*), cg19908812 (*NPY1R*), cg26462130 (*MAD1L1*), cg08904630 (intergenic), cg22799518 (*RBMS2*), cg01744822 (intergenic), and cg15712310 (intergenic)] and two DMPs among males [cg03763518 (*C1orf54*) and cg02042823 (*A2BP1*)] (FDR < 0.05; Table 2). In linear models testing for effect modification by sex, significant Mn × sex interaction terms were observed for cg00954161, cg11161853, cg23903787, cg19908812, cg26462130, cg08904630, cg22799518, cg01744822, cg15712310, and cg03763518 (*p* < 0.05). There was not significant Mn × sex interaction for cg02042823 (*A2BP1*) (*p* = 0.06), although the association between prenatal Mn and DNAm of cg02042823 was nominally significant among females (*B* for each doubling of Mn = 1.08%; *p* = 0.014) but met statistical significance after adjustment for multiple comparisons among males (*B* for each doubling of Mn = 3.40%; FDR = 1.10 × 10^–4^). No DMPs were statistically significant in sex-stratified analyses for other metal exposures (FDR > 0.05), or for As and Hg adjusting for maternal fish intake.

Persistence of cord blood DMPs associated with prenatal Mn and Pb exposure was tested in blood collected in mid-childhood (Table 2). Mn remained persistently associated with greater methylation of cg02042823 (*A2BP1*) among all infants (*B* = 0.75; *p* = 0.01) and for DMPs found in males [cg03763518 (*C1orf54*): *B* = − 3.03; *p* = 1.74 × 10^–4^ and cg02042823 (*A2BP1*): *B* = 1.82; *p* = 2.34 × 10^–4^]. Of the nine Mn-associated DMPs among females, seven were nominally significant in mid-childhood (cg00954161, cg23903787, cg26462130, cg08904630, cg22799518, cg01744822, and cg15712310; *p* < 0.05). Among these CpGs, the direction of association was consistent between the two outcome time points with the exception of cg22799518 (*RBMS2*; cord blood *B* = 2.03, *p* = 2.03 × 10^–7^; mid-childhood *B* = − 0.20, *p* = 0.004). The DMP negatively associated with first-trimester Pb among infants overall (cg20608990; *CASP8*) remained significantly associated in mid-childhood (*B* = − 1.56; *p* = 0.008). Mid-childhood results were consistent when restricting analyses to only children included in the cord blood EWAS (*N* = 189) except for cg22799518 among females, which failed to achieve nominal significance (*p* = 0.07).

We compared our results to previous EWAS of second-trimester maternal RBC Pb (Wu et al. 2017) and Hg (Cardenas et al. 2017) conducted in Project Viva. Among four CpGs associated with second-trimester Pb among infants overall, one was nominally associated with first-trimester Pb in our study (cg22112000: *B* = − 0.09%, *p* = 0.046), and among 38 CpGs associated with second-trimester Pb among females, four were nominally associated with first-trimester Pb in our study (cg17971003: annotated to *SLN*, *B* = 0.90%, *p* = 0.044; cg05959994: *TFDP1*, *B* = − 0.31%, *p* = 2.30 × 10^–4^; cg04571282: *KIAA1267*, *B* = − 0.34%, *p* = 1.11 × 10^–4^; cg11610754: *TMEM59L*, *B* = 0.07%, *p* = 0.039) (Additional file 1: Table S3). The direction of association between Pb and DNAm was consistent between analyses of first- and second-trimester concentrations. Neither of the two CpGs associated with second-trimester Pb among males replicated in our study. One CpG was previously associated with second-trimester Hg among infants overall (cg13340705, *WBP11P1*), and one CpG (cg13416866, *TOR4A*) and one DMR (chr7:94,953,653–94,954,202, *PON1*) was associated with second-trimester Hg among males (Cardenas et al. 2017). Although the CpG associated with Hg among infants overall did not replicate in our study, among males, cg13416866 had a nominally significant association with first-trimester Hg and consistent direction of association (*B* = 0.51%; *p* = 0.028) (Additional file 1: Table S3). Consistent with second-trimester Hg analyses, all CpGs in the region chr7:94,953,653–94,954,202 had a negative direction of association among males in our study of first-trimester Hg, although only one was nominally associated with Hg (cg04871131, *B* = − 0.60; *p* = 0.026) and four had nominal *p* values < 0.10 (cg01874867: *B* = − 1.54; *p* = 0.061; cg17330251: *B* = − 1.55; *p* = 0.051; cg19678392: *B* = − 1.07, *p* = 0.062; cg07404485: *B* = − 0.76, *p* = 0.099).

### Differentially methylated regions

**Fig. 2:**
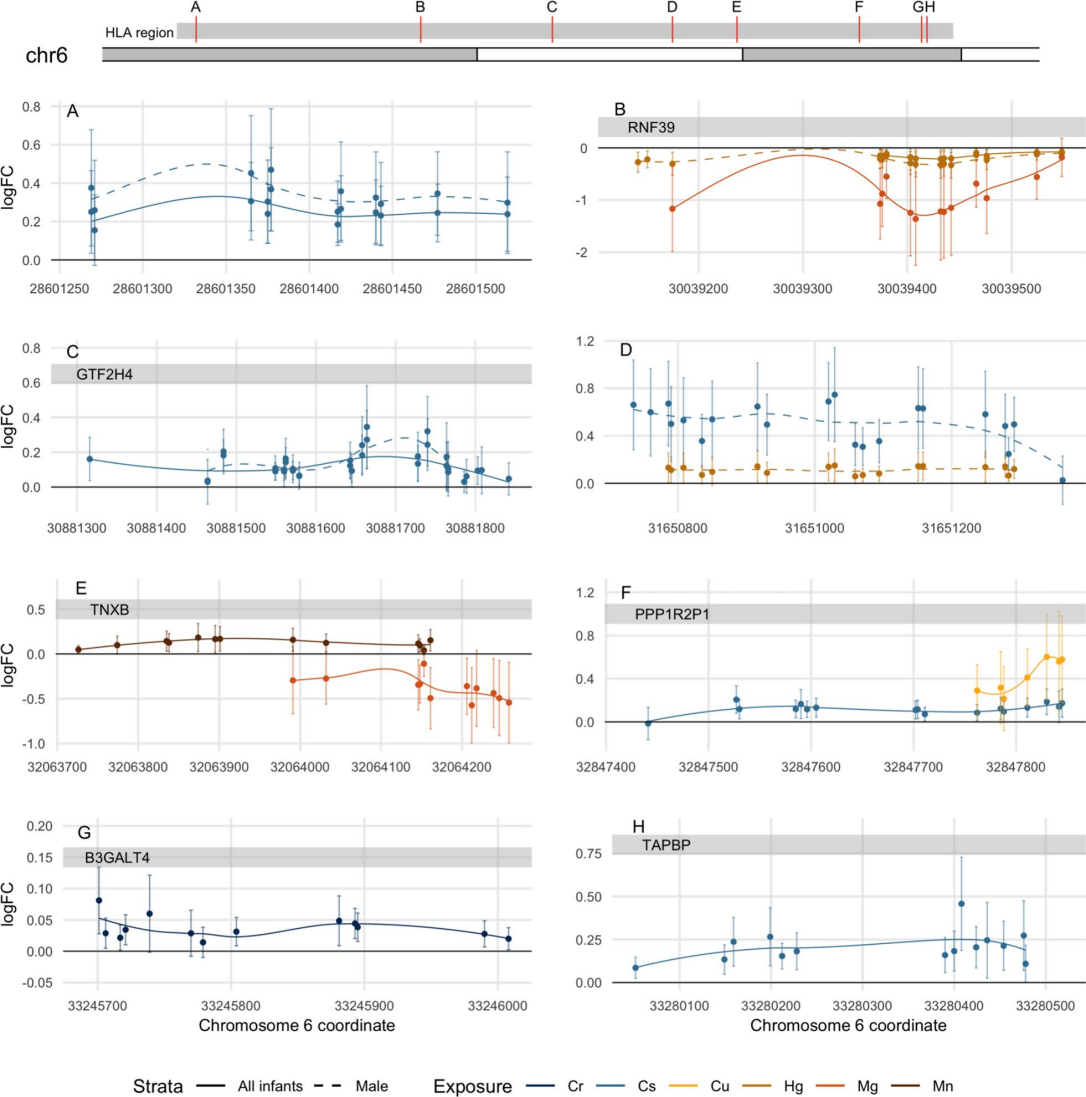
Cord blood differentially methylated regions associated with metals within the human leukocyte antigen region. Cord blood differentially methylated regions (DMRs) located within the human leukocyte antigen (HLA) region (chr6: 28,477,797–33,448,3540) were associated with prenatal exposure to **a** Cr in infants overall and males; **b** Hg in infants overall and males, and Mg in infants overall, **c** Cs in infants overall and males; **d** Cr in females; **e** Cs and Hg in males; **f** Hg and Mn in infants overall; **g** Cs in infants overall; **h** Cr in infants overall; and **i** Cs in infants overall. DMRs identified using *comb-p* (Sidak *p* < 0.05) adjusted for infant sex, race/ethnicity, gestational age, nulliparous, maternal age at enrollment, pre-pregnancy BMI, education, smoking, household income, and estimated cord blood cell-type proportions

The number of DMRs identified in cord blood for each metal among infants overall and stratified by sex is listed in Table 3 (Sidak *p* < 0.05) and is shown in the Manhattan plots in Fig. 1 (noted by bolded individual CpGs within the region). For each DMR, the chromosomal coordinates, gene, probes, probe effect sizes, and *p* values in linear analyses (*limma*) are provided in Additional file 2: Spreadsheets 1–12. In summary, at least one DMR was identified among infants overall for all metals with the exception of Ba and Pb. Cs was associated with the largest number of DMRs among infants overall (*N* = 18). Multiple DMRs associated with Cr, Cs, Cu, Hg, Mg, and Mn were located in the human leukocyte antigen (HLA) region of chromosome 6 (Fig. 2a–h).

**Table 3:** Summary of cord blood differentially methylated regions (Sidak *p* < 0.05) associated with individual metals

	All infants (*N* = 361)		Female (*N* = 169)		Male (*N* = 192)	
	Number of DMRs	Genes	Number of DMRs	Genes	Number of DMRs	Genes
As	1	*LOC144571*	2	*CAT*	3	*LDHC; LOC144571; OXT*
Ba	0		2	*GSDMD; VENTX*	2	*DDX60; LOC619207*
Cd	4	*HOXB7*	2	*HOXA11*	3	*HIST1H1A; SLC17A9*
Cr	3	*B3GALT4; DLC1; TCF7L2*	2	*DLC1*	0	
Cs	18	*ZBTB38; CMYA5; VARS2; GTF2H4; PPP1R2P1; TAPBP; LYNX1; RAD52; PUS7L; IRAK4; ZNF385A; MIR548H4; NOX5; RAI1; SKAP1; SLC44A2; GNAS; GNASAS*	3	*STMN1; GOPC; IRAK4; PUS7L*	14	*ZBTB38; VARS2; GTF2H4; LRRC27; WSCD2; MIR548H4; NOX5; RAI1*
Cu	3	*INO80B; HNRPA1L-2; HNRNPA1; CBX5; GNAS; GNASAS*	1	*GNAS; GNASAS*	6	*NTNG1; PRSS50; HAPLN1; PPP1R2P1; HNRPA1L-2; HNRNPA1; CBX5*
Hg ^a^	2	*RNF39; LYNX1*	0		3	*RNF39; LYNX1*
Mg	5	*FLJ42875; RNF39; TNXB; B4GALNT4; GNAS; GNASAS*	3	*CRIP2; WFIKKN2; GNAS; GNASAS*	1	*AURKC*
Mn	1	*TNXB*	7	*SUCLG2; HLTF; AGPAT4; CARD11; HOXA4; OXGR1*	3	*TTC23; BLCAP; NNAT*
Pb	0		0		2	*SEMA5B*
Se	2	*PF4; GSDMD*	3	*GSDMD; CYP1A1; ADAMTSL5*	1	*AURKC*
Zn	5	*COL7A1; MTNR1B; CACNA2D4; FSCN2; LOC284798*	0		3	*TMEM88B; MCC*

Differentially methylated regions (DMRs) identified using models of individual log_2_-transformed prenatal metal concentrations adjusted for infant sex, race/ethnicity, gestational age, nulliparous, maternal age at enrollment, pre-pregnancy BMI, education, smoking, household income, and estimated cord blood cell-type proportions

^a^All infants: *N* = 358; female: *N* = 167; male: *N* = 191

We assessed the overlap among DMRs identified for each metal (Table 4). Three DMRs were identified as associated with more than one metal in analyses of infants overall for Hg and Mg (chr6: *RNF39*, Fig. 2a), Mg and Mn (chr6: *TNXB,* Fig. 2b), and Cs, Cu, and Mg (chr20: *GNAS*, *GNASAS*).

**Table 4:** Overlapping cord blood differentially methylated regions for metals among infants overall (Sidak *p* < 0.05)

Analysis	Chr	Start	End	Gene	Length	Number of probes
Hg	6	30,039,374	30,039,548	*RNF39*	175	12
Mg		30,039,175	30,039,548	*RNF39*	374	13
Mg	6	32,063,991	32,064,258	*TNXB*	268	12
Mn		32,063,726	32,064,161	*TNXB*	436	13
Cs	20	57,427,274	57,427,762	*GNAS;GNASAS*	489	16
Cu		57,427,426	57,427,942	*GNAS;GNASAS*	517	17
Mg		57,427,170	57,427,973	*GNAS;GNASAS*	804	24

Differentially methylated regions (DMRs) identified using *comb-p* adjusted for infant sex, race/ethnicity, gestational age, nulliparous, maternal age at enrollment, pre-pregnancy BMI, education, smoking, household income, and estimated cord blood cell-type proportions

Results from Gene Ontology (GO) enrichment analysis of DMRs are summarized in Table 5; complete results and differentially methylated genes within GO terms are included in Additional file 2: Spreadsheet 13. We identified nominally significant GO terms with an overrepresentation of differentially methylated genes associated with Cr (*N* = 18), Cs (*N* = 20), Cu (*N* = 17), Se (*N* = 41), and Zn (*N* = 18) (*p* < 0.05); no terms remained significant after FDR adjustment. The majority of GO terms (87%) belonged to the biological process domain. Three GO terms were identified for multiple metals, although differentially methylated genes within GO terms differed between metals. Common GO terms were: cellular response to DNA damage stimulus [GO:00069741, associated with Cs (differentially methylated: *ZBTB38*, *ZNF385A*, *GTF2H4*, *RAD52*) and Cu (*CBX5*, *INO80B*)], response to stress [GO:0006950, Cu (*CBX5*, *GNAS*, *HNRNPA1*, *INO80B*) and Se (*PF4*, *GSDMD*)], and cellular response to stress [GO:0033554; Cs (*ZBTB38*, *ZNF385A*, *GTF2H4*, *IRAK4*, *RAD52*, *NOX5*) and Cu (*CBX5*, *HNRNPA1*, *INO80B*)]. In addition, related terms were identified for multiple metals. For example, regulation of apoptotic process (GO:0042981), regulation of programmed cell death (GO:0043067), and regulation of cell death (GO:0010941) were identified for Cr, whereas cell death (GO:0008219) and programmed cell death (GO:0012501) were identified for Se.

**Table 5:** Summary of Gene Ontology (GO) enrichment analyses of cord blood differentially methylated regions (DMRs)

Cr	Biosynthetic and metabolic processes of glycoproteins, organonitrogen compounds, and carbohydrate derivatives (BP)
	Positive regulation of protein metabolic process (BP)
	Negative regulation of signaling and response to stimulus (BP)
	Regulation of cell death and apoptosis (BP)
	Regulation of cell population proliferation (BP)
	Circulatory system development (BP)
	Protein domain-specific binding (MF)
Cs	Cellular response to stress and oxidative stress (BP)
	Cellular response to DNA damage (BP)
	Organic substance metabolic processes including metabolism of peptides (BP)
	Multicellular organism growth and skeletal system development (BP)
	Protein-DNA complex assembly (BP)
	Regulation of I-kappaB kinase/NF-kappaB signaling (BP)
Cu	Cellular response to stress (BP)
	Cellular response to DNA damage (BP)
	Response to wounding (BP)
	DNA metabolic process (BP)
	Homeostasis and regulation of body fluid levels (BP)
	Viral process (BP)
	Biological process involved in symbiotic interaction (BP)
	Nucleolus and nuclear protein-containing complex (CC)
Se	Response to stress (BP)
	Secretion and vesicle-mediated transport (BP)
	Cell activation (BP)
	Cytokine production and regulation of cytokine production (BP)
	Programmed cell death (BP)
	Immune and inflammatory response (BP)
	Biological process involved in interspecies interaction between organisms (BP)
	Secretion and vesicle-mediated transport (BP)
	Extracellular space (CC)
	Secretory vesicle and vesicle lumen (CC)
Zn	Cation transport (BP)
	Regulation of transmembrane transport (BP)
	Sensory and visual system development (BP)
	System and nervous system process (BP)
	Multicellular organismal signaling (BP)

Prenatal exposure to Cr, Cs, Cu, Se, and Zn was associated with 18, 20, 17, 41, and 18 GO terms, respectively (*p* < 0.05). GO terms were identified for multiple metals are: cellular response to DNA damage stimulus (GO:00069741, associated with Cs and Cu), response to stress (GO:0006950, Cu and Se), and cellular response to stress (GO:0033554; Cs and Cu). Closely related GO terms have been combined; for a complete list of DMR-associated GO terms, see Additional file 2: Spreadsheet 13

*BP* biological process, *CC* cellular component, *MF* molecular function

In sex-stratified analysis, at least one DMR was identified in all analyses among females with the exception of Hg, Pb, and Zn, and in all analyses among males with the exception of Cr (Table 3, Sidak *p* < 0.05). DMRs located in the HLA region were also identified among males (for Cs: chr6: intergenic and *GTF2H4*; for Hg: *RNF39*; Fig. 2a–d). Although the majority of these DMRs were specific to sex-stratified analyses, within metals, we did observe overlap between DMRs identified in analyses of infants overall and stratified by sex (Additional file 1: Table S4). Two sets of overlapping DMRs were associated with multiple metals only in analyses restricted to males: Cs and Hg (chr6: 31,650,735–31,651,361, Fig. 2e) and Mg and Se (chr19: *AURKC*).

To assess persistence of associations within DMRs in mid-childhood, we compared the probe effect sizes and *p* values from DMP analyses within each region between cord blood and mid-childhood (Additional file 2: Spreadsheets 1–12). Among infants overall, four DMRs contained 100% of probes that were nominally significant in mid-childhood (*p* < 0.05), associated with Cd (chr17: *HOXB7*), Mg (chr11: *B4GALNT4*), and Zn (chr17: *FSCN2*; chr20, *LOC284798*). In sensitivity analyses limited to children with cord blood DNAm data, eight DMRs contained 100% of probes that were nominally significant in mid-childhood (*p* < 0.05), associated with Cd (chr2: 200,468,626–200,468,832; chr3: 156,323,952–156,324,118; chr13: 110,319,562–110,319,607; chr17: *HOXB7*), Cs (chr4: 174,421,377–174,422,626; chr12; RAD52), Hg (chr8: *LYNX1*), and Zn (chr12: *CACNA2D4*) (Additional file 2: Spreadsheets 1–12).

## Conclusions

We investigated epigenome-wide associations of prenatal concentrations of 12 metals measured in maternal first-trimester RBC samples with cord blood DNAm and persistence DNAm changes in mid-childhood in the Project Viva pre-birth cohort. Overall we identified two CpGs at which cord blood DNAm was associated with specific metal levels in the first trimester: cg02042823 (*A2BP1*), associated with Mn, and cg20608990 (*CASP8*), associated with Pb, and both DNAm associations seemed to persist in mid-childhood blood. Sex-specific associations with cord blood DNAm at individual CpGs were observed with Mn exposure (9 among females, and 2 CpGs among males that included cg02042823; FDR < 0.05); the two male-specific cord blood DMPs were persistent while six of the female-specific DMPs were persistent in mid-childhood blood DNAm. Multiple DMRs were identified with all prenatal metal exposures among infants overall with the exception of Ba and Pb (Sidak *p* < 0.05). We identified overlapping DNA methylation regions associated with prenatal exposure to Cr, Cs, Cu, Hg, Mg, and Mn in the HLA region. The same cord blood DMR within the *GNAS* gene was associated with prenatal Cs, Cu, and Mg among all infants.

Although our study did not identify individual DMPs associated with exposure to metals other than Mn and Pb, previous EWAS of selected prenatal exposures have identified DMPs. For example, among one of most widely studied metals, As, prior reports in other prenatal cohorts have documented multiple associated DMPs [26, 28–30, 34–36]. These studies used maternal drinking water, urine, or nails to measure As exposure, which capture toxic inorganic forms of As. Urinary As in these studies was measured as the sum of inorganic species, whereas As is primarily present in inorganic forms in nail samples [37]. In our study, we used maternal RBCs which provide total arsenicals, including less toxic organic forms. In addition, the majority of previous studies have been conducted in populations with moderate-to-high As exposure, whereas exposure was low in our study. These factors potentially explain the lack of significant associations for our study. An EWAS of prenatal maternal blood Cd concentrations and DNAm in cord blood in a Bangladeshi cohort (*N* = 127) identified sex-specific effects more pronounced in boys, although no DMPs were statistically significant after adjustment for multiple comparisons [38]. This is in line with our study in which there was no evidence of DMPs associated with prenatal blood Cd levels. In our study median cadmium levels were much lower (0.39 ng/g) compared to median levels in the Bangladeshi study (1.3 ug/kg).

Mn is an essential trace element necessary for numerous biological functions including the metabolism of amino acids, proteins, lipids, and carbohydrates; immune function; energy regulation; and protection against free radicals [39]. Mn is also necessary for brain development and cognitive function, although Mn is a neurotoxicant at excessive levels [40], and in epidemiological studies, high levels of exposure to Mn prenatally have been negatively associated with measures of neurodevelopment [8, 41]. Sex-specific associations have also been observed between prenatal Mn exposure and adolescent neuromotor [42] and neurobehavioral function [43], with high exposure associated with overall poorer outcomes among females compared with males. Few EWAS have previously investigated associations between prenatal Mn exposure and DNAm in offspring. An EWAS of placental DNAm and infant toenail Mn concentrations in a US birth cohort (*N* = 61) identified differential methylation at five CpGs with tertiles of Mn exposure (*p*_Bonferroni_ < 0.05) [44]; however, no Bonferroni-significant DMPs nor DMPs at *p* < 1 × 10^–4^ overlapped with the Mn-associated DMPs or DMRs identified in our study. Given the sensitive window of prenatal development, timing of exposure and tissue used for Mn measurement could influence findings which might explain the lack of overlap in study results.

We identified Mn-associated DMPs annotated to genes with roles in neurodevelopment. Among infants overall and in stratified analyses of male infants, we found Mn exposure to be associated with greater methylation of cg02042823, annotated to Ataxin-2-binding protein 1 (*A2BP1*), also known as RNA binding fox-1 homolog 1 (*RBFOX1*). A2BP1 is an RNA binding protein involved in tissue-specific pre-mRNA splicing of transcripts involved in neuronal development [45, 46]. *A2BP1*-knockout mice have splicing changes in proteins involved in synaptic transmission and increased neuronal excitability [47]. In humans, *A2BP1* variants have been associated with biomarkers of Alzheimer’s disease [48] and copy number variation of *A2BP1* has been associated with autism spectrum disorder [49]. We also found prenatal Mn exposure among females to be associated with methylation of cg19908812, annotated to Neuropeptide Y receptor Y1 (*NPY1R*), which encodes a transmembrane protein involved in neurotransmitter activity. NPY1R expression has been associated with anxious temperament in monkeys [50] and deletion involving *NPY1R* has been identified in a case study of autism [51]. Similarly, cg26462130, annotated to Mitotic arrest deficient 1 like 1 (*MAD1L1*), was associated with Mn in female infants. MAD1L1 is involved in mitotic spindle-assembly and genetic variation of *MAD1L1* has been associated with schizophrenia [52] and anxiety disorders [53]. Whether our findings are implicated in neurodevelopmental disruption remains to be determined.

Prenatal Pb exposure has been negatively associated with mental and cognitive development and sensory function in infants and children [54–56]. In a previous study conducted in the Project Viva cohort, where Pb levels were below the CDC reference of 5 μg/dL, there was a trend in lower neurobehavioral measures with higher prenatal Pb exposure [57]. Animal models have provided evidence that Pb effects global DNAm levels via DNA methyltransferase inhibition [58, 59], and prenatal Pb exposure has been negatively associated with global levels DNAm measured in cord blood [60]. There is limited research on epigenome-wide associations between Pb and locus-specific association with DNAm; however, an EWAS conducted in a Mexican pre-birth cohort (*N* = 420) identified no associations between prenatal exposure to Pb and cord blood DNAm [31]. Overall, we similarly found null results in Pb analyses with the exception of the DMP cg20608990, annotated to Caspase 8 (*CASP8*). CASP8 is involved in cell apoptosis [61], including neuronal apoptosis [62, 63], and cell death may be induced by Pb through a pathway involving CASP8 [64]. DNAm of the *CASP8* promoter was not found to be associated with Pb and Cd exposure among children environmentally exposed through e-waste recycling (*N* = 116), although this study differed from our analyses in age participants (i.e., exposure and DNAm measured at 3–7 years old) [65]. In addition, this study measured DNAm at four loci within the *CASP8* promoter using pyrosequencing.

In Project Viva, previous EWAS have been conducted for second-trimester maternal RBC Pb (*N* = 268) [32] and second-trimester maternal RBC Hg [33] (*N* = 321). Using a look-up approach of CpGs associated with second-trimester Pb, in our study first-trimester Pb was nominally associated with one CpG among all infants and four CpGs among females (*p* < 0.05), with a consistent direction of association across EWAS. In the EWAS of second-trimester Hg, DNAm of cg13340705 (*WBP11P1*) and cg13416866 (*TOR4A*) was positively associated with exposure among infants overall and males, respectively (*p*_Bonferroni_ < 0.05) [33]. In the current analyses of first-trimester RBC Hg concentrations, cg13416866 had a nominally significant positive association with Hg among males. In addition, Cardenas et al. identified one DMR associated with second-trimester Hg among male infants: chr7:94,953,653–94,954,202, annotated to Paraoxonase 1 (*PON1*) and consisting of 9 CpGs with negative associations with Hg (FDR < 0.05). Similarly, all CpGs in this region had a negative direction of association among males in our study of first-trimester Hg, although only one was nominally associated with Hg (cg04871131, *p* = 0.026). Limited replication of results between EWAS of first- and second-trimester exposures may be due to windows of susceptibility to epigenetic dysregulation or incomplete overlap between mother–infant pairs included in first-trimester and second-trimester EWAS of Hg and Pb in Project Viva. These results highlight the complexity of epigenetic dysregulation for trimester-specific exposures during fetal development, a period of rapid epigenetic reprogramming.

Our study found multiple DMRs located in the HLA region associated with Cr, Cs, Hg, Mg, and Mn, including common DMRs associated with Hg and Mg (annotated to *RNF39*) and Mg and Mn (annotated to *TNXB*). The HLA region spans a ~ 4 Mb segment of the chromosomal band 6p21 and contains an overrepresentation of protein-coding genes [66]. The HLA region is involved in immune regulation, including inflammation and innate and adaptive immunity, as well as reproduction, central nervous system development, and neurological disorders [66]. Ring Finger Protein 29 (*RNF39*) is a non-HLA expressed gene involved in regulation of synaptic plasticity [67]. RNF39 was found to be differentially expressed in rodent brains exposed to methylmercury [68], and differential methylation of *RNF39* has been associated with schizophrenia spectrum disorders [69] and post-traumatic stress disorder [70]. Tenascin XB (*TNXB*) is also a non-HLA expressed gene, and *TNXB* methylation has similarly been associated with psychiatric disorders including social anxiety disorder [71] and anorexia nervosa [72]. We also identified multiple DMRs annotated to *GNAS* associated with Cs, Cu, and Mg among all infants, and with Cu and Mg among females. GNAS complex locus (*GNAS*) is a protein-coding imprinted gene which includes four alternative exons. Methylation of *GNAS* has been associated with schizophrenia spectrum disorders [69], and parent-or-origin effects of SNPs have been associated with DNAm of *GNAS* [73]. In GO enrichment analysis of cord blood DMRs, Cs and Cu were nominally associated with GO terms including *GNAS* and involved in a broad range of biological processes including growth, development, response to stress, and metabolic processes.

In addition, GO analyses of DMRs identified similar terms nominally associated with multiple prenatal metals. Specifically, terms related to response to stress were enriched for Cs-, Cu-, and Se-associated differentially methylated genes; terms related to apoptosis and cell death were enriched for Cr- and Se-associated differentially methylated genes; and terms related to macromolecule metabolic processes were enriched for Cr-, Cs-, and Cu-associated differentially methylated genes. Interestingly, differentially methylated genes in related pathways differed between metals, suggesting that prenatal metal exposures may affect common biological pathways through epigenetic regulation of multiple genes.

This study was strengthened by the use of maternal blood metal concentrations measured in the first trimester, which served as an unbiased biomarker of prenatal exposure in early life. We were also able to confirm reliability of maternal blood metal concentrations by calculating ICCs using duplicate samples. Overall, metal concentration measurements had good reliability (ICCs > 0.70); however, Cr and Cu had lower ICCs (0.40 and 0.64, respectively), which should be considered in interpreting the results from these EWAS. In addition, our study measured multiple metals in maternal blood samples and DNAm of offspring at birth and in mid-childhood. This allowed us to compare EWAS results across prenatal metal exposures in the same population, as well as test for the persistence of differential methylation in childhood. Although our sample size was moderate, we were able to conduct stratified analyses to investigate sex-specific effects of metal exposures. It should be noted, however, that we adjusted for multiple comparisons within each metal EWAS (i.e., 394,460 probes), and we did not adjust for the number of metals tested or multiple testing introduced in sex-stratified analyses, which may increase the number of false-positives. This study was also strengthened by analyzing both locus-specific and regional differential methylation. DMRs were identified using the *comb-p* algorithm, which has been shown to have higher power and greater ability to identify DMRs with small effect sizes than other methods to detect DMRs [74] but at the expense of a higher Type I error rate [75]. Therefore, our results reflect candidate genomic positions associated with prenatal metal exposure, and the robustness of our findings should be tested in other cohorts and confirmed in meta-analyses. Many of the DMRs we identified were restricted to sex-stratified analyses, suggesting sexual dimorphism in the epigenomic effects of prenatal metal exposures; however, further research is needed to understand sex-specific regional differences in DNAm as most studies limit analyses across sex.

Several limitations of our study should be considered. Maternal blood metal exposure was assessed at a single time, and a single measurement may not capture critical windows of vulnerability during fetal development to epigenetic dysregulation. We also lacked data and samples for gene expression and cannot determine if changes in DNAm were associated with alterations in gene expression. In addition, although we investigated persistence of metal-induced epigenetic changes in mid-childhood, we did not have measures of childhood metal exposure which may also impact epigenetic programming. Most Mn-associated DMPs were nominally significant in mid-childhood (*p* < 0.05 for all Mn-associated DMPs among infants overall and males, and 78% of DMPs among females); however, we observed limited persistence of DMRs in mid-childhood (four DMRs had *p* < 0.05 in 100% of probes in mid-childhood). We are unable to assess how metal exposure between birth and mid-childhood may have affected DNA methylation levels. Our analyses of persistence of effects in mid-childhood were also limited by the incomplete overlap between mother–infant pairs with cord blood and mid-childhood DNAm data (*N* = 189 mother–infant pairs with data at both time points). Furthermore, we chose to analyze associations between each metal exposure and DNAm separately to identify independent effects on epigenetic dysregulation. However, prenatal metal exposure may also act as a mixture to affect DMAm profiles. DNA methylation disruption across the genome resulting from prenatal maternal exposures appear non-targeted with multiple studies finding evidence of numerous associations that appear cohort specific. This might indicate the importance of taking into account environmental mixtures in epigenetic research. Statistical methods are needed in order to capture the complexity of environmental mixtures on the epigenome.

Few studies have investigated effects of multiple prenatal metal exposures on epigenome-wide DNAm and its persistence in childhood. In this study of 12 metals, we provide evidence that prenatal metal exposure is associated with cord blood DNAm at individual CpGs for two specific metals (Mn and Pb). In addition, we observed associations between metal exposures and overlapping DMRs annotated to genes related to neurodevelopment and co-located in the HLA region, although analyses should be replicated in other cohorts to determine the generalizability of our findings. Further research may also provide insights to the effect of mixtures of prenatal metal exposures on epigenetic dysregulation as well as the impact of changes in DNAm on gene expression and biological pathways and child health outcomes.

## Methods

### Study design

Project Viva is a longitudinal pre-birth cohort established to examine associations between maternal diet and other environmental factors and maternal and child health. The Project Viva cohort has previously been described in detail [76]. Briefly, we recruited women between 1999 and 2002 from Atrius Harvard Vanguard Medical Associates, a group practice in eastern Massachusetts, US Research staff screened women at their initial obstetric visit (median 9.9 weeks of gestation). Exclusion criteria were multiple gestation, not English-speaking, ≥ 22 weeks gestation, and plan to leave the study area prior to delivery. Initially, we included a total of 2,670 pregnancies (64% of those screened), of which 2,128 live births remained in the cohort at the time of delivery (Additional file 1: Figure S1). For mothers with two children included in the current dataset (*N* = 2), we excluded the second birth from the current analyses.

At study recruitment (median = 10 weeks gestation), research assistants administered a brief interview and provided mothers with a questionnaire to return by mail. Research assistants also conducted a mid-pregnancy visit, a hospital visit during the birth admission, and visits during infancy and mid-childhood (median 7.66 years). At birth, research assistants collected cord blood samples. Mothers provided written informed consent at enrollment and at the mid-childhood visit. Protocols for biosample collection were designed to minimize discomfort and inconvenience for mothers and children. The Institutional Review Board of Harvard Pilgrim Health Care reviewed and approved all study protocols.

### Sample collection, processing, and analysis

*Maternal RBC metal analysis:* We collected blood samples from pregnant women at recruitment. We centrifuged blood samples (2,000 rpm for 10 min at 4 °C) to separate erythrocytes from plasma and stored aliquots at -70 °C prior to analyses. We performed sample handling in an ISO class 6 clean room with an ISO class 5 laminar flow clean hood. Briefly, 0.5 ml of packed erythrocytes was weighed and digested in 2 ml of ultra-pure concentrated HNO_3_ acid for 48 h, and then further digested with 1 ml of 30% ultra-pure hydrogen peroxide for 24 h before diluting to 10 ml with deionized water.

We measured first-trimester concentrations of the metals aluminum (Al), As, Ba, Cd, cobalt (Co); Cr, Cs, Cu, Mg, Mn, molybdenum (Mo); nickel (Ni), Pb, antimony (Sb), Se, tin (Sn), Tl: thallium, vanadium (V), and Zn in erythrocytes using a triple quadrupole inductively coupled plasma mass spectrometry (ICP-MS) (Agilent 8800 ICP-QQQ) on a single run in MS/MS mode using appropriate cell gases and internal standards. We analyzed Hg concentrations separately using a Direct Mercury Analyzer 80 (Milestone Inc.). For Hg, three samples had insufficient quantity for analysis.

We applied the following quality control (QC) measures: analysis of initial and continuous calibration verification, procedural blanks, and repeated analysis of 2% of samples. In addition, Seronorm-Blood L3 was analyzed daily as QC samples with one blinded sample at high and low levels run per batch. Recoveries of QC standards were between 90 and 110% with the exception of Al, Sn, and Sb where measured values were below the limit of detection (LOD) out of the acceptable range 80–120%. Intraday CVs were calculated using three concentrations of in-house QC pools (*n* = 7). Intraday CVs were < 5% for all analytes with the exception of Se and Sb, which were < 10%. Interday CVs for all elements were < 15% except for concentrations near the LOD. We measured metals concentrations as ng/g red blood cells. In this analysis, we include only metals that met the quality control criteria of percent detected ≥ 80% and intraclass correlation coefficients (ICCs) ≥ 0.70 among duplicates, with the exception of Cr and Cu which had ICCs = 0.40 and 0.64, respectively, but had detectable measures in over 80% of the samples.

*Infant and child blood and DNAm analysis*: Clinicians collected cord blood at delivery using a syringe and needle from the umbilical vein. Paper or electronic “flags” on participants’ charts were used to prompt clinical staff to collect samples, and cord blood samples were collected from approximately 75% of participants who delivered at the study hospitals. We collected fasting blood samples from children at the mid-childhood visit using ethylenediaminetetraacetic acid (EDTA)-containing vacutainer tubes, and put samples on ice. To separate plasma, nucleated cells (including leukocytes and nucleated RBCs in cord blood and leukocytes in child blood), and RBCs, we centrifuged the tubes at 1,700×g for 10 min at 4 °C within 24 h of collection.

Measurement of DNAm has previously been described [32]. Briefly, we extracted genomic DNA from the nucleated cells with PureGene Kits (Fisher, Catalog Nos. A407-4, A416-4; Qiagen, Catalog Nos. 158908, 158912, 158924), and we stored sample aliquots at − 80 °C until analysis. Research staff performed bisulfite conversion using the Zymo DNA Methylation kit (Zymo Research, Irvine, CA). One μg DNA of each sample was randomized across plates and BeadChips to reduce batch effects. DNAm analysis was performed at Illumina, Inc. using the Illumina Infinium HumanMethylation450 (450K) BeadChip (Illumina, San Diego, CA). The 450K microarray interrogates > 485,000 methylation loci at single-nucleotide resolution.

### Covariates

We collected maternal demographics (including education and household income), smoking status, and pre-pregnancy height and weight through self-administered questionnaires and interviews during pregnancy. We estimated maternal fish intake as servings per week for the first trimester or period closest to blood draw using a semi-quantitative food frequency questionnaire [77]. We used maternal self-reported height and weight to calculate pre-pregnancy body mass index (BMI; kg/m^2^). We estimated gestational age from mothers’ last menstrual period (LMP) at enrollment; we used gestational age determined by ultrasound if available and differed from LMP by > 10 days [76].

### Data processing

DNAm data were preprocessed using the R package *minfi* [78]. We excluded duplicate samples, samples with low individual call rates (< 0.98), and samples that had a genotype or sex mismatch (*N* = 22 cord blood DNAm; Additional file 1: Figure S1). Non-CpG probes and probes with detection *p* values > 0.05 for > 1% of samples were dropped. In addition, we excluded probes located on sex the chromosomes, cross hybridizing probes [79], and probes associated with single-nucleotide polymorphisms (SNPs) that have a minor allele frequency ≥ 0.05 at the single base extension or within the target region. A total of 394,460 high-quality probes remained for analyses. We performed background correction and dye-bias equalization using the normal–exponential out-of-band method (noob) [80], and probe-type normalization using the beta-mixture quantile method (BMIQ) implemented through *minfi*. We estimated cell-type composition using the Houseman regression calibration method [81] implemented in the *minfi* package [78] using reference panels derived from cord blood nucleated cells for cord blood cell-type estimates [82] and adult leukocytes for mid-childhood cell-type estimates [83].

### Data analysis

Metal concentrations < the LOD were replaced with LOD/√2. The LODs and the number of samples < LOD for each metal were: As: 0.153 ng/g (*N* = 35); Ba: 0.412 ng/g (*N* = 5); Cd: 0.0569 ng/g (*N* = 19): Cr: 0.685 ng/g (*N* = 37); Cs: 0.0587 ng/g (*N* = 0); Cu: 1.85 ng/g (*N* = 0); Hg: 0.3 ng/g (*N* = 9); Mg: 4.15 ng/g (*N* = 0); Mn: 0.422 ng/g (*N* = 0); Pb: 0.0746 ng/g (*N* = 0); Se: 1.73 ng/g (*N* = 0); Zn: 8.74 ng/g (*N* = 0)]. Prior to analyses, we log_2_ transformed metal concentrations to meet model assumptions as metals were right skewed.

We presented descriptive statistics for participant characteristics and metal concentration using medians and interquartile ranges (IQRs) for continuous variables and frequencies and proportions for categorical variables. We assessed differences between mother–infant pairs with cord blood and mid-childhood DNAm data using the Mann–Whitney test for continuous variables and Chi-squared test for categorical variables. We tested associations between prenatal first-trimester metal concentrations and estimated cord blood cell-type proportions [B cells, CD4 + T cells, CD8 + T cells, granulocytes, monocytes, natural killer (NK) cells, and nucleated RBCs] with linear models adjusted for infant sex, race/ethnicity, gestational age, nulliparous, maternal age at enrollment, pre-pregnancy BMI, education (< college graduate or college graduate), household income (≤ $70,000 per year or > $70,000 per year), and maternal smoking (never, former, or smoking during pregnancy).

We conducted EWAS for DMPs (i.e., individual CpGs) using linear regression with empirical Bayes smoothing of standard errors with the robust estimator for prior variances implemented using the R package *limma* [84]. To better meet models assumptions, we performed analyses using the logit-transformation of Beta-values (i.e., M-value = ln[Beta-value / (1—Beta-value)]) [85]. We conducted EWAS using *limma* independently for each log_2_-transformed prenatal metal concentration (i.e., 12 EWAS of cord blood DNAm). Overall, we adjusted cord blood models for infant sex, race/ethnicity, gestational age, nulliparous, maternal age at enrollment, pre-pregnancy BMI, education, household income, maternal smoking, and estimated cell-type distribution (B cells, CD4 + T cells, CD8 + T cells, granulocytes, monocytes, NK cells, and nucleated RBCs). For each analysis, we generated Q-Q plots and calculated the genomic inflation factor (λ) to evaluate potential systematic biases. Within each metal EWAS, we used the Benjamini–Hochberg false discovery rate (FDR) method [86] to adjust for multiple comparisons using the *p.adjust* function in R. To address potential effect modification by infant sex, we performed *limma* analyses for infants overall and stratified by sex. For FDR-significant probes in sex-stratified analyses, we further evaluated effect modification using linear models including a metal × sex interaction term. We performed sensitivity analysis for associations with As and Hg including the covariate of maternal fish consumption during pregnancy. We report DMP effect sizes on the Beta-value scale for interpretability.

We analyzed DMRs using the *comb-p* method [87] implemented in the R package *ENmix* [88]. *Comb-p* identities DMRs using all EWAS *p* values and associated chromosomal coordinates by first adjusting for autocorrelation between nearby probes using the Stouffer–Liptak–Kechris (*slk*) correction (e.g., *p* values with adjacent low values will be pulled lower). An FDR correction is applied to *slk*-adjusted *p* values, regions are identified among probes meeting a specified FDR threshold, and a single *slk*-adjusted *p* value is calculated for each region. Correction for multiple comparisons is performed on regional *p* values using a Sidak correction based on the total number of possible regions. We performed *comb-p* using the arguments of a maximum distance to combine DMRs of 1,000 base pairs, the recommended bin size for autocorrelation of 310, and the FDR significance threshold of 0.001. Regions containing only one probe were excluded, and regions with Sidak *p* < 0.05 were considered significant. GO enrichment analysis of DMRs was performed using *goregion* implemented in the R package *missMethy* [89, 90]. The *goregion* function uses genes annotated to DMRs to identify GO terms including an overrepresentation of differentially methylated genes while accounting for coverage of genes on the 450K microarray [91]. *goregion* was implemented independently for each metal with the input of cord blood DMRs (Sidak *p* < 0.05) and all CpGs included in analyses. REVIGO was used to aid in interpretation of relationships between identified GO terms [92].

To test the persistence of observed associations between prenatal metal exposure and cord blood DNAm, we performed *limma* analyses with mid-childhood DNAm. Models included the covariates of infant sex, race/ethnicity, child age at blood draw, nulliparous, maternal age at enrollment, pre-pregnancy BMI, education, household income, maternal smoking, and mid-childhood estimated cell-type composition (B cells, CD4 + T cells, CD8 + T cells, granulocytes, monocytes, and NK cells). We performed sensitivity analyses restricting samples to children included in the cord blood DNAm analyses. We used a nominal *p* value < 0.05 along with consistency of direction of association to evaluate persistence of associations in mid-childhood.

To compare our findings of results of previous EWAS of second-trimester maternal RBC Hg and Pb in Project Viva [32, 33], we used a look-up approach of CpGs identified as associated with Hg and Pb by Wu et al. and Cardenas et al., respectively. We used an unadjusted *p* value < 0.05 and direction of association to determine consistency of results between EWAS of first- and second-trimester metal exposures.

We performed all data analysis using R 4.0.3. [93].

## Supplementary Information

**Additional file 1**

**Additional file 2**

## Data Availability

Datasets generated and analyzed during the current study are not publicly available because we did not obtain consent for such public release of epigenetic data from participants. However, raw data to generate figures and tables are available from the corresponding author with the appropriate permission from the Project Viva study team and investigators (project_viva@hphc.org) upon reasonable request and institutional review board approval. R code for all analyses is available at the study’s GitHub repository (https://github.com/annebozack/viva_DNAm_metals). Complete cord blood EWAS results for all metals are available at GitHub repository and Open Science Framework (OSF) site (https://osf.io/jf5yt/).

## Abbreviations

Cu: Copper
Mg: Magnesium
Mn: Manganese
Se: Selenium
Zn: Zinc
As: Arsenic
Cd: Cadmium
Pb: Lead
Hg: Mercury
DOHaD: Developmental origins of health and disease
DNAm: DNA methylation
EWAS: Epigenome-wide association study
DMPs: Differentially methylated positions
US: United States
RBCs: Red blood cells
Ba: Barium
Cr: Chromium
Cs: Cesium
DMRs: Differentially methylated regions
LOD: Limit of detection
IQR: Interquartile range
DMPs: Differentially methylated positions
DMRs: Differentially methylated regions
Al: Aluminum
Co: Cobalt
Mo: Molybdenum
Ni: Nickel
Sb: Antimony
Sn: Tin
Tl: Thallium
V: Vanadium
ICP-MS: Inductively coupled plasma mass spectrometry
QC: Quality control
LOD: Limit of detection
ICC: Intraclass correlation coefficients
EDTA: Ethylenediaminetetraacetic acid
450K microarray: Illumina Infinium HumanMethylation450 BeadChip
FFQ: Food frequency questionnaire
BMI: Body mass index
LMP: Last menstrual period
SNPs: Single-nucleotide polymorphism
noob: Normal–exponential out-of-band
BMIQ: Beta-mixture quantile
IQRs: Interquartile ranges
NK: Natural killer cells
FDR: False discovery rate
slk: Stouffer–Liptak–Kechris
A2BP1: Ataxin-2-binding protein 1
GO: Gene ontology
RBFOX1: RNA binding fox-1 homolog 1
NPY1R: Neuropeptide Y receptor Y1
MAD1L1: Mitotic arrest deficient 1 like 1
CASP8: Caspase 8
RNF39: RING finger protein 39
TNXB: Tenascin XB
GNAS: GNAS complex locus
PON1: Paraoxonase 1
