# Supplementary material for: Prenatal metal exposure, cord blood DNA methylation and persistence in childhood: an epigenome-wide association study of 12 metals
Source: Clin Epigenetics. 2021 Nov 19;13:208. doi: 10.1186/s13148-021-01198-z (PMC8605513; doi:10.1186/s13148-021-01198-z)
Supplement: Supplementary file 1 — Additional file 1 [file 13148_2021_1198_MOESM1_ESM.docx]

**ADDITIONAL MATERIAL**

**Title:** Prenatal metal exposure, cord blood DNA methylation and persistence in childhood: an epigenome-wide association study of twelve metals

**Authors:** Anne K. Bozack, Sheryl L. Rifas-Shiman, Brent A. Coull, Andrea A. Baccarelli, Robert O. Wright, Chitra Amarasiriwardena, Diane R. Gold, Emily Oken, Marie-France Hivert, Andres Cardenas

**Additional Figure 1: Flowchart of participant enrollment and study inclusion**

**
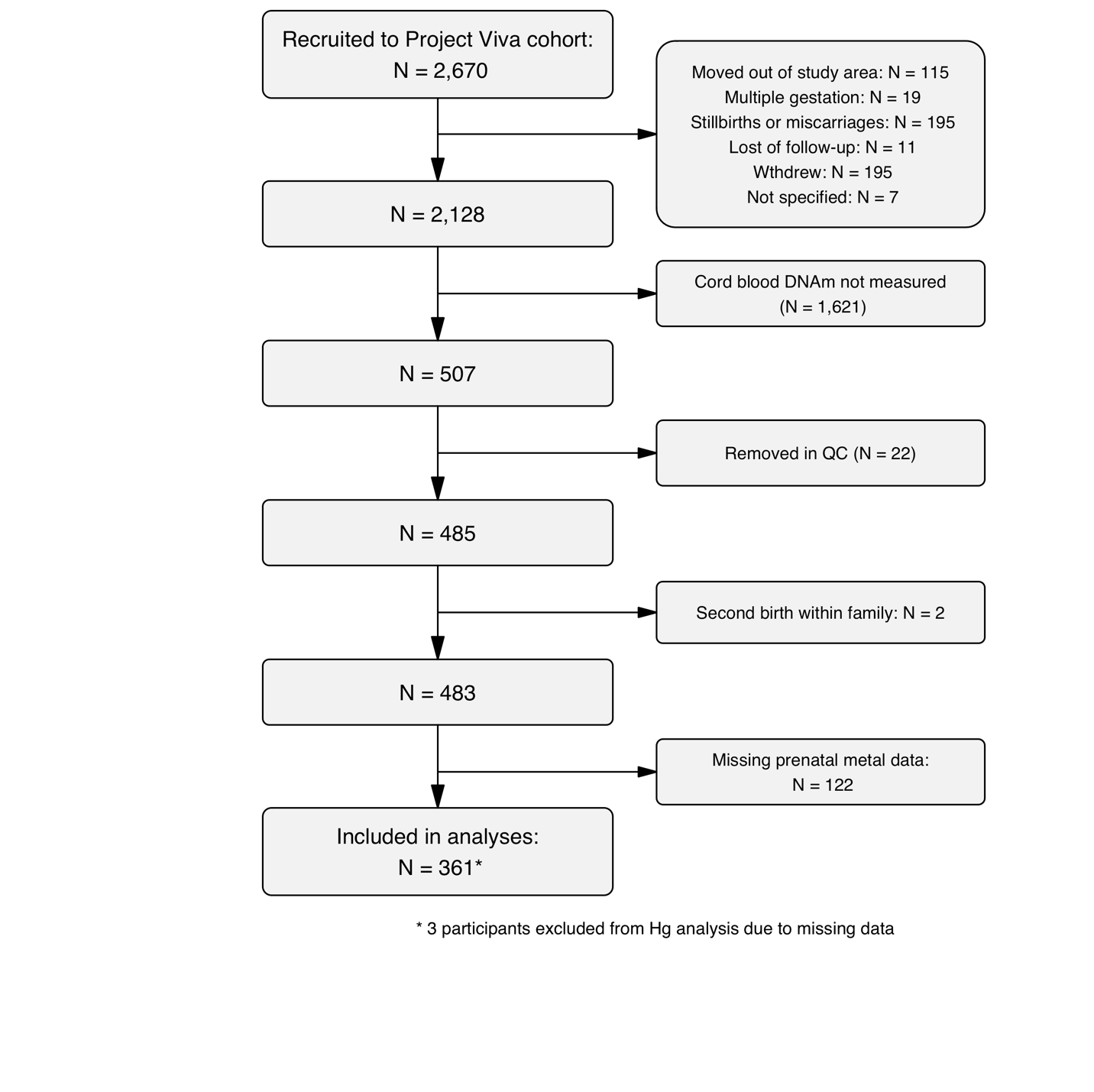
**

**Additional Figure 2: Spearman correlations between prenatal first trimester metals measured in red blood cells (N = 361). * *p* < 0.05.**

**
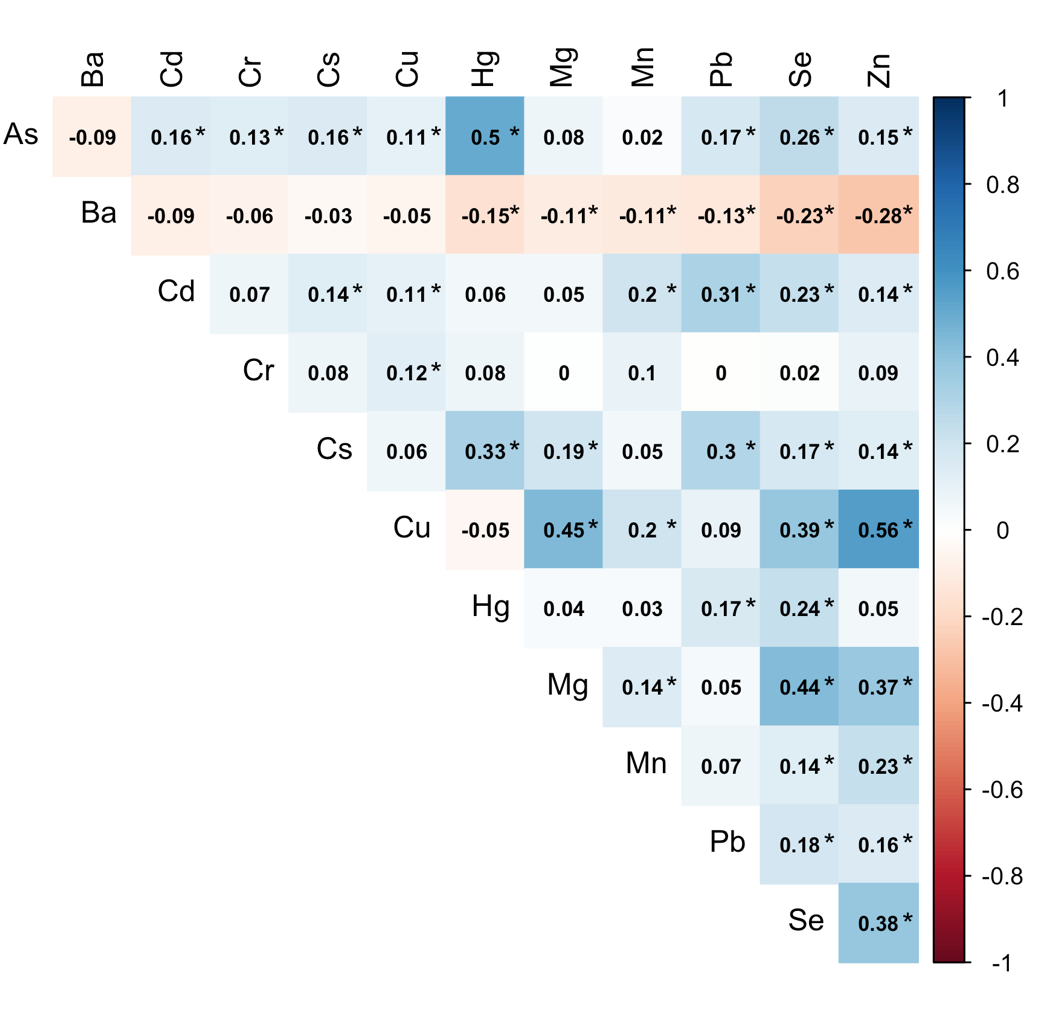
**

**Additional Table 1: Associations between prenatal metal exposure and cell type abundance percentages in cord blood.** Results from linear models for log_2_-transformed metal concentrations adjusted for infant sex, race/ethnicity, gestational age, nulliparous, maternal age at enrollment, pre-pregnancy BMI, education, smoking, and household income. Significant associations (*p* < 0.05) are bolded.

|  | **B cells** | | **CD4+ T cells** | | **CD8+ T cells** | | **Granulocytes** | | **Monocytes** | | **NK cells** | | **Nucleated RBCs** | |
| --- | --- | --- | --- | --- | --- | --- | --- | --- | --- | --- | --- | --- | --- | --- |
| **Metal** | ***B*** | ***p*** | ***B*** | ***p*** | ***B*** | ***p*** | ***B*** | ***p*** | ***B*** | ***p*** | ***B*** | ***p*** | ***B*** | ***p*** |
| As | 0.16 | 0.16 | -0.04 | 0.88 | 0.02 | 0.86 | -0.26 | 0.54 | 0.20 | 0.10 | 0.01 | 0.91 | -0.06 | 0.87 |
| Ba | -0.17 | 0.18 | -0.20 | 0.46 | -0.16 | 0.26 | 0.75 | 0.10 | -0.16 | 0.23 | -0.02 | 0.84 | -0.05 | 0.90 |
| Cd | 0.08 | 0.56 | 0.63 | 0.05 | 0.03 | 0.87 | -0.40 | 0.45 | 0.08 | 0.58 | -0.13 | 0.29 | -0.34 | 0.41 |
| Cr | -0.19 | 0.25 | 0.13 | 0.72 | -0.16 | 0.37 | 0.47 | 0.42 | -0.03 | 0.85 | -0.18 | 0.20 | -0.02 | 0.97 |
| Cs | -0.02 | 0.97 | -0.03 | 0.97 | 0.01 | 0.99 | -0.57 | 0.64 | 0.49 | 0.16 | 0.20 | 0.50 | -0.12 | 0.90 |
| Cu | -0.67 | 0.35 | **3.25** | **0.037** | -0.90 | 0.27 | -1.18 | 0.65 | -1.22 | 0.10 | -0.43 | 0.49 | 0.47 | 0.82 |
| Hg | -0.05 | 0.64 | -0.33 | 0.19 | 0.01 | 0.96 | 0.49 | 0.23 | 0.23 | 0.05 | 0.04 | 0.65 | -0.36 | 0.28 |
| Mg | -0.23 | 0.70 | -0.75 | 0.57 | -0.33 | 0.62 | 2.10 | 0.33 | -0.74 | 0.23 | -0.29 | 0.58 | -0.10 | 0.95 |
| Mn | 0.02 | 0.91 | -0.16 | 0.73 | 0.35 | 0.13 | -0.41 | 0.58 | 0.10 | 0.66 | -0.07 | 0.70 | 0.22 | 0.71 |
| Pb | 0.18 | 0.55 | 0.95 | 0.14 | -0.46 | 0.16 | -1.91 | 0.07 | 0.09 | 0.78 | 0.27 | 0.28 | 0.77 | 0.36 |
| Se | 0.01 | 0.99 | 1.15 | 0.37 | 0.46 | 0.49 | -1.50 | 0.48 | -0.37 | 0.55 | 0.31 | 0.55 | -0.46 | 0.79 |
| Zn | 0.21 | 0.75 | 2.08 | 0.15 | -0.84 | 0.26 | 1.04 | 0.66 | -1.05 | 0.12 | -0.51 | 0.37 | -1.76 | 0.35 |
| NK = natural killer; RBCs = red blood cells | | | | | | | | | | | | | | |

**Additional Table 2: Genomic inflation factors (λ) for associations between prenatal metal exposure and cord blood DNAm.** Models of individual log_2_-transformed prenatal metal concentrations adjusted for infant sex, race/ethnicity, gestational age, nulliparous, maternal age at enrollment, pre-pregnancy BMI, education, smoking, household income, and estimated cord blood cell type proportions.

|  | **All infants (N = 361)** | **Female (N = 169)** | **Male (N = 192)** |
| --- | --- | --- | --- |
|  | **λ** | **λ** | **λ** |
| As | 1.04 | 0.95 | 1.01 |
| Ba | 1.01 | 0.80 | 0.86 |
| Cd | 0.91 | 0.95 | 0.97 |
| Cr | 1.01 | 0.84 | 0.92 |
| Cs | 1.02 | 0.78 | 1.09 |
| Cu | 1.84 | 1.05 | 1.92 |
| Hg ^a^ | 0.80 | 0.71 | 0.79 |
| Mg | 1.07 | 0.87 | 1.60 |
| Mn | 0.74 | 0.89 | 0.84 |
| Pb | 0.91 | 1.11 | 0.88 |
| Se | 0.87 | 0.72 | 1.01 |
| Zn | 1.48 | 1.04 | 1.10 |
| a. All infants: N = 358; female: N = 167; male: N = 191. λ = genomic inflation factor. | | | |

**Additional Figure 3: Q-Q plots for epigenome-wide associations between prenatal metal exposure and differential methylation in cord blood.** DMPs identified using *limma* models of log_2_-transformed prenatal metal concentrations adjusted for infant sex, race/ethnicity, gestational age, nulliparous, maternal age at enrollment, pre-pregnancy BMI, education, smoking, household income, and estimated cord blood cell type proportions.


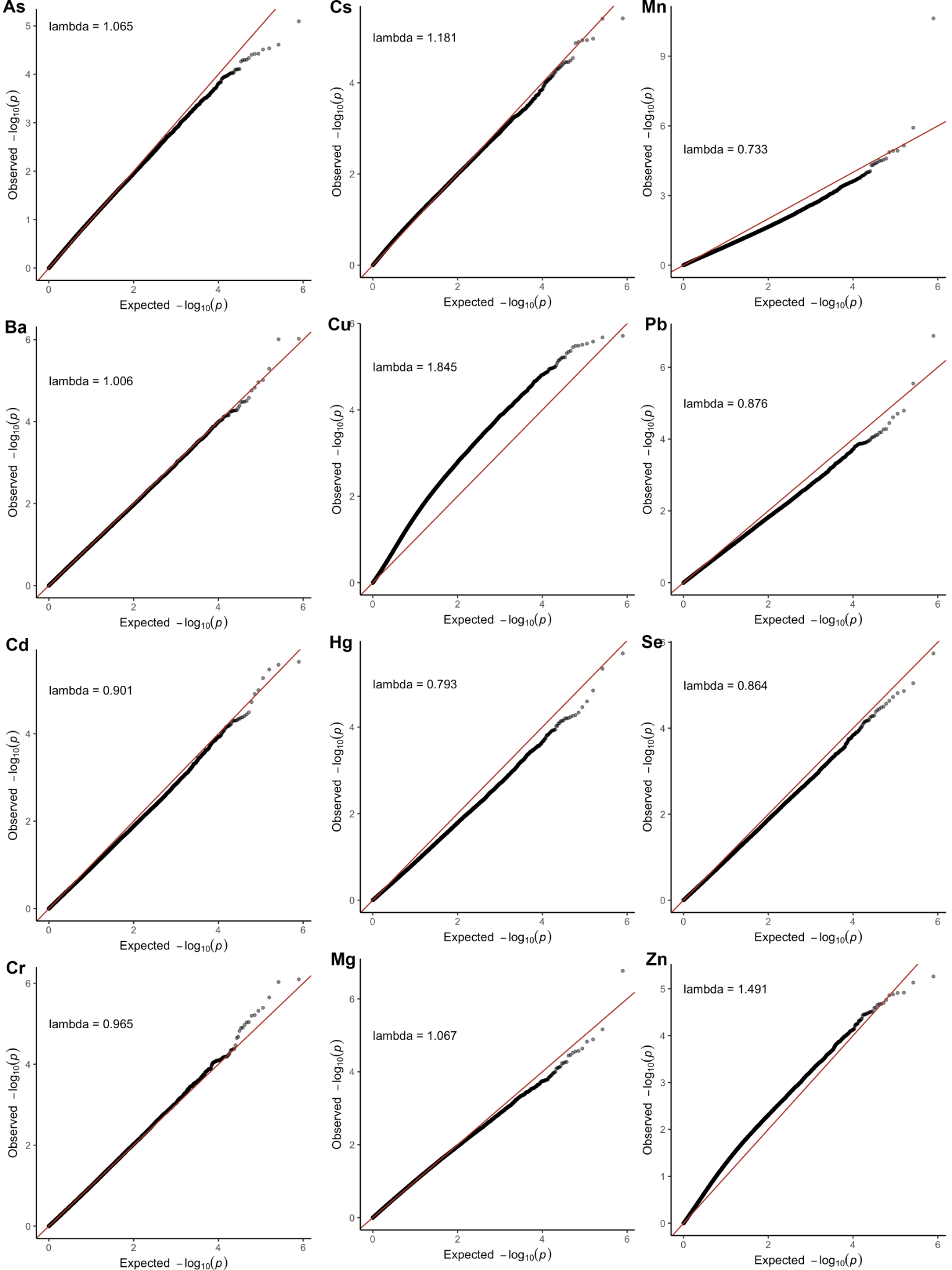


**Additional Table 3: Lookup of cord blood CpGs previously reported to be associated with second-trimester maternal RBC Pb (*N* = 268; *FDR* < 0.05) (Wu et al. 2017) and Hg (*N* = 321; *p_Bonferroni_* < 0.05 and *FDR* < 0.05 for DMPs and DMRs, respectively) in Project Viva.** In current study, *p*-values from models of individual log_2_-transformed prenatal metal concentration adjusted for infant sex (if not stratified), race/ethnicity, gestational age, nulliparous, maternal age at enrollment, pre-pregnancy BMI, education, smoking, household income, and estimated cord blood cell type proportions. Mean difference in % methylation determined using the same adjusted models for Beta-values for interpretation of results.

| **Associations with Pb** | | | | **Wu et al.** | | **Present study** | |
| --- | --- | --- | --- | --- | --- | --- | --- |
| **CpG** | **Chr** | **Position** | **Gene** | **Mean difference in % methylation^a^** | ***p*-value** | **Mean difference in % methylation^a^** | ***p*-value** |
| **All infants** |  |  |  |  |  |  |  |
| cg02272457 | chr6 | 42,427,790 | – | -0.1 | 1.2×10^−6^ | -0.1 | 0.11 |
| cg20324491 | chr7 | 149,128,397 | – | -0.5 | 8.3×10^–8^ | -1.9 | 0.15 |
| cg22112000 | chr7 | 5,647,182 | – | -0.1 | 1.1×10^−7^ | **-0.9** | **0.046** |
| cg10773601 | chr19 | 51,226,046 | *CLEC11A* | -1.4 | 2.3×10^−7^ | -0.8 | 0.08 |
| **Male infants** |  |  |  |  |  |  |  |
| cg22512536 | chr5 | 154,134,507 | *LARP1* | 0.1 | 2.2×10^−7^ | 0.1 | 0.07 |
| cg08964024 | chr12 | 14,923,326 | – | 0.1 | 2.2×10^−8^ | 0.1 | 0.06 |
| **Female infants** | |  |  |  |  |  |  |
| cg04295372 | chr1 | 101,184,332 | *VCAM1* | 1.6 | 3.9×10^−6^ | 0.4 | 0.66 |
| cg09356083 | chr1 | 2,430,057 | *PLCH2* | -0.6 | 6.9×10^−7^ | -0.2 | 0.48 |
| cg20816789 | chr1 | 27,462,916 | *SLC9A1* | -0.8 | 3.8×10^−7^ | 0.2 | 0.50 |
| cg22446399 | chr2 | 27,326,580 | *CGREF1* | -0.5 | 4.2×10^−6^ | -0.5 | 0.09 |
| cg27403609 | chr2 | 11,101,403 | – | -1.2 | 3.4×10^−4^ | -0.6 | 0.14 |
| cg07248017 | chr4 | 8,230,689 | *SH3TC1* | -1.0 | 1.2×10^−6^ | 0.0 | 0.96 |
| cg06239355 | chr5 | 32,714,010 | *NPR3* | 0.1 | 1.5×10^−7^ | 0.0 | 0.71 |
| cg15601915 | chr5 | 1,065,775 | *SLC12A7* | -0.9 | 1.6×10^−6^ | -0.5 | 0.27 |
| cg17599748 | chr6 | 31,589,597 | *SNORA38* | -1.4 | 1.1×10^−6^ | -0.1 | 0.88 |
| cg08673909 | chr7 | 1,329,592 | – | -0.2 | 6.3×10^−6^ | -0.1 | 0.48 |
| cg10090217 | chr7 | 45,151,583 | *TBRG4* | -0.1 | 5.0×10^−6^ | 0.0 | 0.96 |
| cg16565528 | chr8 | 91,010,920 | – | 1.8 | 7.9×10^−7^ | 0.3 | 0.54 |
| cg03152353 | chr9 | 139,417,194 | *NOTCH1* | -1.1 | 4.6×10^−7^ | -0.1 | 0.90 |
| cg13817920 | chr9 | 96,587,843 | – | -0.6 | 4.4×10^−5^ | -0.1 | 0.61 |
| cg13791644 | chr10 | 119,794,366 | *RAB11FIP2* | 1.0 | 1.3×10^−5^ | 0.2 | 0.58 |
| cg18454045 | chr10 | 88,391,658 | – | -1.3 | 2.8×10^−7^ | -0.8 | 0.17 |
| cg11127561 | chr11 | 125,462,151 | *STT3A* | 0.1 | 4.6×10^−5^ | 0.1 | 0.11 |
| cg17971003 | chr11 | 107,582,804 | *SLN* | 1.0 | 1.7×10^−6^ | 0.9 | **0.044** |
| cg24637308 | chr11 | 6,592,297 | *DNHD1* | -4.3 | 1.1×10^−6^ | -2.0 | 0.10 |
| cg05959994 | chr13 | 114,253,916 | *TFDP1* | -0.2 | 1.9×10^−8^ | -0.3 | **2.30×10^−4^** |
| cg11203293 | chr13 | 25,777,762 | – | -2.3 | 3.4×10^−7^ | -0.6 | 0.45 |
| cg04545835 | chr14 | 102,675,570 | *WDR20* | -0.4 | 4.0×10^−5^ | -0.2 | 0.15 |
| cg08131309 | chr15 | 40,399,131 | *BMF* | -1.6 | 6.2×10^−7^ | -1.3 | 0.15 |
| cg04730825 | chr16 | 16,116,191 | *ABCC1* | -1.7 | 3.8×10^−6^ | 0.2 | 0.66 |
| cg26686608 | chr16 | 88,705,716 | *IL17C* | 1.2 | 1.7×10^−6^ | 1.1 | 0.06 |
| cg00461015 | chr17 | 42,295,635 | *UBTF* | -0.1 | 1.7×10^−5^ | -0.1 | 0.13 |
| cg04571282 | chr17 | 44,108,753 | *KIAA1267* | -0.2 | 4.8×10^−6^ | -0.3 | **1.11×10^−4^** |
| cg11252953 | chr17 | 80,358,829 | *C17orf101* | 0.8 | 8.7×10^−7^ | 0.2 | 0.70 |
| cg15922057 | chr17 | 25,784,714 | – | -1.2 | 8.9×10^−6^ | -0.1 | 0.85 |
| cg06753949 | chr19 | 15,334,309 | – | -0.2 | 7.8×10^−6^ | 0.0 | 0.53 |
| cg07780528 | chr19 | 35,630,334 | *FXYD1* | -0.8 | 2.0×10^−6^ | -0.5 | 0.21 |
| cg11610754 | chr19 | 18,722,595 | *TMEM59L* | 0.1 | 3.1×10^−6^ | 0.1 | 0.39 |
| cg18598117 | chr19 | 941,126 | *ARID3A* | -1.5 | 2.8×10^−5^ | -0.3 | 0.90 |
| cg22217660 | chr19 | 50,765,301 | *MYH14* | -0.7 | 1.2×10^−5^ | 0.3 | 0.27 |
| cg03373781 | chr20 | 30,582,113 | *XKR7* | -1.1 | 2.3×10^−7^ | 0.0 | 0.90 |
| cg07341934 | chr20 | 57,463,711 | *GNAS* | -1.5 | 2.2×10^−6^ | 0.3 | 0.69 |
| cg21307155 | chr20 | 3,216,740 | *SLC4A11* | -0.8 | 8.1×10^−8^ | -0.5 | 0.28 |
| cg17174023 | chr22 | 50,987,453 | *KLHDC7B* | -1.0 | 3.1×10^−5^ | -0.8 | 0.13 |
| **Associations with Hg** | | | | **Cardenas et al.** | | **Present study** | |
| **All infants** |  |  |  |  | |  | |
| **CpG** | **Chr** | **Position** | **Gene** | **Mean difference in % methylation^a^** | ***p*-value** | **Mean difference in % methylation^a^** | ***p*-value** |
| cg13340705 | 18 | 30,091,235 | WBP11P1 | 0.3 | 5.3×10^−7^ |  | 0.88 |
| **Male infants** |  |  |  |  |  |  |  |
| cg13416866 | 9 | 140,175,679 | *TOR4A* | 1.3 | 3.5×10^−8^ | 0.5 | **0.028** |
| cg07404485^b^ | 7 | 94,953,653 | *PON1* | -2.2 | 7.2×10^−4^ | -0.8 | 0.10 |
| cg05342682 | 7 | 94,953,680 | *PON1* | -1.8 | 8.0×10^−3^ | -0.4 | 0.32 |
| cg04155289 | 7 | 94,953,770 | *PON1* | -1.7 | 3.3×10^−3^ | -0.5 | 0.20 |
| cg19678392 | 7 | 94,953,810 | *PON1* | -2.9 | 8.5×10^−4^ | -1.1 | 0.06 |
| cg21856205 | 7 | 94,953,877 | *PON1* | -1.6 | 9.9×10^−4^ | -0.5 | 0.16 |
| cg17330251 | 7 | 94,953,956 | *PON1* | -3.7 | 1.6×10^−3^ | -1.6 | 0.05 |
| cg01874867 | 7 | 94,954,059 | *PON1* | -3.8 | 8.2×10^−4^ | -0.5 | 0.06 |
| cg20119798 | 7 | 94,954,144 | *PON1* | -2.7 | 2.5×10^−5^ | -0.8 | 0.15 |
| cg04871131 | 7 | 94,954,202 | *PON1* | -1.0 | 1.5×10^−4^ | -0.6 | **0.026** |
| a. Change in % methylation for each doubling of maternal Pb or Hg concentrations. b. CpGs located in the second-trimester Hg-associated DMR chr7:94,953,653-94,954,202. | | | | | | | |

**Additional Table 4: Overlapping differentially methylated regions (DMRs) identified in sex-stratified analyses (Sidak *p* < 0.05).** DMRs identified using *comb-p* adjusted for infant sex (if not stratified), race/ethnicity, gestational age, nulliparous, maternal age at enrollment, pre-pregnancy BMI, education, smoking, household income, and estimated cord blood cell type proportions.

| **Analysis** | **Chr** | **Start** | **End** | **Gene(s)** | **Length** | **Number of probes** |
| --- | --- | --- | --- | --- | --- | --- |
| Cd, all infants | 2 | 200,468,626 | 200,468,832 |  | 207 | 3 |
| Cd, female |  | 200,468,626 | 200,468,728 |  | 103 | 2 |
| Cs, all infants; Cs, male | 3 | 141,087,187 | 141,087,363 | *ZBTB38* | 177 | 5 |
| Cs, all infants; Cs, male | 6 | 28,601,269 | 28,601,519 |  | 251 | 11 |
| Hg, all infants | 6 | 30,039,374 | 30,039,548 | *RNF39* | 175 | 12 |
| Hg, male |  | 30,039,142 | 30,039,548 | *RNF39* | 407 | 15 |
| Cs, all infants | 6 | 30,881,316 | 30,881,842 | *VARS2;GTF2H4* | 527 | 21 |
| Cs, male |  | 30,881,464 | 30,881,766 | *VARS2;GTF2H4* | 303 | 15 |
| Cs, male | 6 | 31,650,735 | 31,651,362 |  | 628 | 21 |
| Hg, male |  | 31,650,786 | 31,651,291 |  | 506 | 18 |
| Cs, all infants | 6 | 32,847,441 | 32,847,845 | *PPP1R2P1* | 405 | 17 |
| Cs, male |  | 32,847,762 | 32,847,845 | *PPP1R2P1* | 84 | 7 |
| Cr, all infants; Cr, female | 8 | 13,373,033 | 13,373,141 |  | 109 | 3 |
| Cs, all infants | 8 | 43,131,260 | 43,131,656 |  | 397 | 5 |
| Cs, male |  | 43,131,260 | 43,132,507 |  | 1248 | 8 |
| Hg, all infants; Hg, male | 8 | 143,859,669 | 143,859,990 | *LYNX1* | 322 | 7 |
| Se, all infants; Ba, female | 8 | 144,635,260 | 144,635,610 | *GSDMD* | 351 | 9 |
| Se, female |  | 144,635,260 | 144,636,113 | *GSDMD* | 854 | 12 |
| As, all infants; As, male | 12 | 9,217,390 | 9,217,859 | *LOC144571* | 470 | 9 |
| Cs, all infants; Cs, female | 12 | 44,152,509 | 44,152,940 | *IRAK4;PUS7L* | 432 | 11 |
| Cu, all infants; Cu, male | 12 | 54,673,867 | 546,74,009 | *CBX5;HNRNPA1;HNRPA1L-2* | 143 | 4 |
| Cs, all infants; Cs, male | 15 | 69,325,271 | 69,325,560 | *MIR548H4;NOX5* | 290 | 5 |
| Cs, all infants | 17 | 17,603,531 | 17,603,837 | *RAI1* | 307 | 4 |
| Cs, male |  | 17,603,531 | 17,604,146 | *RAI1* | 616 | 5 |
| Cu, all infants |  | 57,427,426 | 57,427,942 | *GNAS;GNASAS* | 517 | 17 |
| Cu, female |  | 57,427,443 | 57,427,942 | *GNAS;GNASAS* | 500 | 16 |
| Mg, all infants |  | 57,427,170 | 57,427,973 | *GNAS;GNASAS* | 804 | 24 |
| Mg, female |  | 57,427,443 | 57,427,762 | *GNAS;GNASAS* | 320 | 13 |
| Mg, male | 19 | 57,742,112 | 57,742,423 | *AURKC* | 312 | 7 |
| Se, male |  | 57,742,255 | 57,742,423 | *AURKC* | 169 | 6 |
| Cu, all infants |  | 57,427,426 | 57,427,942 | *GNAS;GNASAS* | 517 | 17 |
| Cu, female |  | 57,427,443 | 57,427,942 | *GNAS;GNASAS* | 500 | 16 |
| Mg, all infants |  | 57,427,170 | 57,427,973 | *GNAS;GNASAS* | 804 | 24 |
| Mg, female |  | 57,427,443 | 57,427,762 | *GNAS;GNASAS* | 320 | 13 |
